# Miconazole-Urea in a Buccal Film as a New Trend for Treatment of Resistant Mouth Fungal White Patches

**DOI:** 10.3389/fmicb.2018.00837

**Published:** 2018-05-11

**Authors:** Omar Y. Mady, Ahmed M. Donia, Lamiaa A. Al-Madboly

**Affiliations:** ^1^Department of Pharmaceutical Technology, Faculty of Pharmacy, Tanta University, Tanta, Egypt; ^2^Department of Pharmaceutical Microbiology, Faculty of Pharmacy, Tanta University, Tanta, Egypt

**Keywords:** bioadhesive film, *C. albicans*, miconazole, urea, MIC

## Abstract

A growing concern about *Candida albicans* is the emergence of high incidence of resistance against antifungal agents, which requires searching for new medications or improving the response to the existing members. The objective of this work was to evaluate the effectiveness of the miconazole in the absence and presence of urea, as a penetration enhancer, against *C. albicans*. In addition to, formulating both of them in a polymer film to be used topically for treatment of mouth fungal white patches caused by *C. albicans.* A synergistic effect was recorded between this imidazole and urea against the test strain. Miconazole MIC (32 mg/L) was dramatically reduced to 0.0625 mg/L following combination with urea. Transmission electron microscopy explained the mechanisms of action mediated by the test agents. Minimal fungicidal dose of miconazole combined with urea showed early apoptotic cells with condensed chromatin and small blebs. Cytoplasmic leakage and necrosis in some cells was observed at high fungicidal dose. Buccal bioadhesive films were prepared using increasing values of the drug MIC and urea. The physicochemical characters of the prepared films including; film weight, thickness, swelling index, drug content, folding endurance, surface pH, bioadhesion force and time and drug release kinetics, were studied. Microbiological evaluation of all prepared films showed an increase in the inhibition zone diameters for films containing increasing concentrations of both miconazole and urea in a concentration-dependent manner (30–40 mm) compared to miconazole alone (18 mm). Based on our results, the prepared films are promising for buccal administration of miconazole/urea showing synergistic effect for treatment of *C. albicans* infection.

## Introduction

Oral candidiasis is a leading cause of morbidity and mortality in patients suffering from cancer (Albougy and Naidoo, 2002). The treatments are based on using antimycotic therapy in high doses, which is undesirable because of the potential adverse effects. To diminish these effects as well as the risk of drug resistance emergence, topical therapy candidate should be the first choice for treating both oral and pharyngeal candidiasis. The efficiency of antifungal therapy depends on the contact time and the drug level that should be above the minimum inhibitory concentration (MIC) (Roey et al., 2004).

Miconazole is an imidazole derivative that has a wide spectrum of activity against dermatophytes and yeast. It is effective against systemic candidiasis but with higher toxicity compared to other azoles that is why it is restricted to topical preparations (Martindale and Westcoot, 2007). Accordingly, there is a need to improve the total biological activity of this agent with other substances to convert it from fungistatic to fungicidal. Literature reported some trials for combining miconazole with other drugs, which may be either synthetic or natural in origin. In combining with miconazole, a synergistic interaction was reported with a secondary plant metabolite namely polygodial (Kubo et al., 2011), griseofulvin (Mahmoudabadi et al., 2006), and fluconazole (Mikami et al., 1992).

Delivery of drugs through buccal cavity is an innovative research area for the systemic delivery of orally inactive drugs. The delivery of drugs through the oral mucosa can be categorized into three groups. First is sublingual administration in which drugs reach the systemic circulation through contact with the mucous membranes beneath the tongue. Second, buccal delivery, in which the drug administration occurs via the buccal mucosa lining the cheeks. Third, local drug delivery in which the drug is delivered into the oral cavity giving a local effect. Mucoadhesion is a term used for materials bind to the mucin layer of the biological membranes. It is considered a key element for buccal drug delivery systems development. Buccal adhesive drug delivery systems include films, matrix tablets, layered systems, microspheres, disks, hydrogel and ointments (Martin et al., 2003; Sudhakar et al., 2006; Patel et al., 2007; Vishnu et al., 2007). Of these systems, buccal films are preferred over tablets because of the patient comfort, high accuracy and long lasting time compared with semisolids (Verma et al., 2011).

Propylene glycol is Generally Recognized As Safe (GRAS) by the Food and Drug Administration (FDA) and is widely used in pharmaceutical (buccal films) and food preparations (Murray and George, 1997). The study conducted by Jhundoo et al. (2015) revealed absence of significant cytotoxic effects of propylene glycol on the three tested cell lines namely epithelial cells (HT29-MTX), endothelial cells (HUVEC), and macrophage (RAW264.7). Arechabala et al. (1999) tested the effect of different surfactants including; tween 80, texapon N40, tween 60, texapon K1298, triton 3100, and benzethonium chloride on the viability of normal human fibroblasts using the neutral red test, MTT assay and lactate dehydrogenase release. They found that tween 80 was the least cytotoxic agent. Furthermore, Barabas et al. (2008) mentioned that urea did neither induce cytokine secretion from PMNCs nor activate the maturation of dendritic cells, and hence, it had neither cytotoxic nor immune activity characters in the cultured cells. Therefore, the aim of this study was to develop and assess a buccal film for miconazole delivery in the absence and the presence of urea at different MICs.

## Materials and Methods

### Materials

Miconazol base and carboxymethyl cellulose were obtained as donated samples from Sigma Pharmaceutical Company, Quesna, Egypt. Polyvinyl alcohol (PVA, M- 31000 g/mol) was purchased from Carl Roth GmbH (United Kingdom). Tween 80 (TW) and carboxymethylcellulose (CMC) were bought from El-Nasr Pharmaceutical Chemicals Co., Cairo, Egypt. Propylene glycol (PG) was purchased from BDH chemical Ltd. (Poole, England).

### Test Microorganism

*Candida albicans* (MTCC 227) the test strain was obtained from Department of Microbiology, Faculty of Pharmacy, Tanta University, Tanta, Egypt.

### Methods

#### Antifungal Susceptibility Testing of Miconazole Against *C. albicans*

The MIC of miconazole, in the absence and presence of urea, was performed by microtiter dilution assay according to the standard method of the European Committee on Antimicrobial Susceptibility Testing (EUCAST-E. DEF 7.3, 2015). Sterile, plastic microtiter trays with 96 flat-bottom wells were utilized. A double strength RPMI 1640 medium with L-glutamine and 2% glucose was prepared for proper 50% dilution after inoculum addition. Miconazole was dissolved in dimethylsulfoxide (DMSO) as a stock solution. Appropriate diluted working solutions (2× of the final required concentration) of miconazole, urea, and both of them were prepared in double strength RPMI with 1% DMSO, according to EUCAST (E. DEF 7.3, 2015). Wells 1–10 of each column (row A) were filled with 100 μl of each prepared miconazole concentration which was double the final required concentration (2×). About 100 μl of urea working solutions were transferred to the corresponding wells of row B to evaluate its effect on the test organism. Combinations of different miconazole concentrations and a fixed concentration of urea (10%) were also tested in a similar way (row C). Control wells (column 11) contained 100 μl of drug-free medium. Sterility control wells (column 12) were filled with 100 μl of sterile distilled water.

*Candida albicans* was the test organism grown on Sabouraud’s dextrose agar for 24 ± 2 h in ambient air at 35 ± 2°C prior to testing. A suspension of an overnight grown test organism was prepared in sterile distilled water until the turbidity matched that of a 0.5 McFarland standard that was equivalent to 1-5x10^6^ CFU/ml. This suspension was further diluted 1 in 10 to yield a final working suspension of 1–5 × 10^5^ CFU/ml. A volume of 100 μl was taken from the later yeast suspension and then transferred to each well in the plate, without touching its content, to achieve an inoculum density of 0.5–2.5 × 10^5^ CFU/ml. Viability counts were carried out for purposes of quality control to ensure the proper well density. Following incubation at 35 ± 2°C for 24 ± 2 h, plates were read at 530 nm using TECAN Sunrise^TM^ microdilution plate reader (Austria). Blank value was subtracted from the readings of the other wells. The MIC was defined as the lowest concentration, in mg/L, of the test drug alone or in combination at which, growth inhibited by ≥50% as compared to the drug-free control. Values of the minimum fungicidal concentration (MFC) were calculate by subculturing aliquots from all wells showed negative growth into Sabouraud agar then incubated as previously mentioned above. All experiments were carried out in triplicates and the mean values were calculated for the MIC and MFC in addition to the standard deviation SD. Breakpoints of miconazole against *C. albicans* were *R* > 8 mg/L and *S* ≤ 1 mg/L (Richter et al., 2005; Hanafy and Morsy, 2012, EUCAST break points table, ver. 9, 2018; http://www.eucast.org/fileadmin/src/media/PDFs/EUCAST_files/AFST/Clinical_breakpoints/Antifungal_breakpoints_v_9.0_180212.pdf). The fractional inhibitory concentration (FIC) was calculated through dividing the MIC of miconazole combined with urea by the MIC of miconazole alone. The FIC value was used to interpret the interaction outcome as follows: synergism (FIC ≤ 0.5), additive (1 ≥ FIC > 0.5), indifferent (4 ≥ FIC > 1), and antagonism (FIC > 4) as described by Kubo and Lee (1998).

#### Transmission Electron Microscopy

Test samples were initially prepared for electron microscopy at the Microbiology Laboratory, Department of Pharmaceutical Microbiology, Faculty of Pharmacy, Tanta University, Egypt. *C. albicans* was treated with a sub MIC of 16 mg/L of miconazole, 10% urea or a combination of both 10% urea and low fungicidal dose (0.016 mg/L) or high fungicidal dose (0.031 mg/L) of miconazole in RPMI medium for 24 h at 37°C. Cells were collected by centrifugation at 4000 rpm for 15 min. The pellet was washed twice with phosphate buffered saline (PBS, pH 7). Next, samples were dispersed in the fixative solution and sent to the Electron Microscope Unit, Faculty of Agriculture, Al-Mansoura University for transmission electron microscopy (TEM).

#### Preparation of Buccal Film

Solvent casting method was used for preparation of the buccal films (Mahajan et al., 2011). A clear solution was formed due to dissolution of polymers in hot water. The required amount of either urea, miconazole or both was dissolved in the prepared polymer solution. Next, propylene glycol and tween 80 were transferred to the mixture. The solution casting was performed in a Petri dish (78.6 cm^2^) and dried in oven at 40°C. Last, dry films were cut in the form of square shaped sections (4 cm^2^).

#### Evaluation of the Prepared Buccal Film

Weight uniformity was determined gravimetrically according to Semalty et al. (2008). The individual weight of each sample (4 cm^2^) from each film was determined by the used of an electrical balance. The mean and standard deviation were calculated. The thickness of the samples was measured by Vernier Caliper (Giradkar et al., 2010). Analysis of the results for mean and standard deviation was performed. Repeated folding of the film at the same site until breaking or folded up to 200 times with no breaking was used for determination of folding endurance (Khairnar et al., 2009). The medicated film theoretically expected to contain one or more of the determined MIC of miconazole base was dissolved in 100 ml phosphate buffer pH 6.8. The prepared solution was diluted with the same buffer before spectrophotometric quantification of the drug concentration at a aaa of 272 nm (Hiremath et al., 2009). The actual drug content was expressed in mg/4 cm^2^. The palatability of the film was ensured by determining the microenvironment pH of the developed buccal films. A combined glass electrode was utilized. At ambient temperature, films (4 cm^2^) were soaked in 5 ml of distilled water for an hour. The surface pH was determined after equilibration for a minute using electrode mounting on the surface of the swollen film (Khairnar et al., 2009). Triplicate experiments were performed.

#### Swelling Index

The film sample (1 cm^2^) was put in a pre-weighed special holder of a dissolution apparatus. The loaded holder was weight at zero time before submerging in buffer solution, then taken out and reweighed after careful removal of any excess solution. The increase in the film weight was recorded at time intervals (5, 10, 15, 20, 30, 40, and 60 min). The experiment was conducted until a fixed weight was recorded. Calculation of the swelling index was done using the following formula (Koland et al., 2012):

$$\text{Swelling index}=(W_{t}-W_{0})/W_{0}$$

Where *W*_t_ represents the film weight at time *t* and *W*_0_ is the film weight at zero time.

#### *In Vitro* Bioadhesion Strength

The rabbit intestine was used as a model mucosal membrane for measuring the bioadhesive strength of the prepared film, (Nafee et al., 2003). First, rabbits were scarified and the excised intestine was washed gently with phosphate buffer (pH 6.6). To expose the mucosal surface, the intestine was cut longitudinally and then cut into rectangular parts (4 cm^2^) that were fixed on the surface of a holder made of cellulose acetate plastic film using cyanoacrylate adhesive so that the mucosal surface is uppermost. The film (4 cm^2^) was pasted to another holder of similar size. The intestinal surface was wetted with phosphate buffer (pH 6.6). The two holders carrying the intestine and the film were placed in contact with each other with fixed light pressure between fingers for a minute (preload time) to ease the adhesion bonding. The upper tissue holder was hanged on an iron stand with the aid of an aluminum wire fixed with a hook fastened on the back of the holder.

A polypropylene bag, which is pre-weighted, was fixed to the hook on the backside of the lower film holder using an aluminum wire. Following 1 min, water was transferred to the polypropylene bag by an adjustable burette to deliver water at constant rate (2.0 drops/second) until detachment of the film from the tissue. The water collected in the bag was weighed and expressed as the weight (g) needed for the detachment (bioadhesive strength) (Alanazi et al., 2007). The adhesion force of bond strength was determined according to the following equations (Habib et al., 2010):

Adhesion Force (*N*) = (Bioadhesive strength (g) × 9.81)/1000

Bond strength (*N* m^-2^) = Force of adhesion/film surface area.

#### *In Vitro* Bioadhesion Time

The residence time of the films was evaluated, *in vitro*, by recording the time needed for these films to detach from the intestinal mucosa (Singh et al., 2008). The intestine was fastened with mucosal side facing up on the surface of a glass slide with cover slips by the aid of cyanoacrylate glue. Mucosa was wetted with phosphate buffer solution (pH 6.6).

The film (1 cm^2^) was moistened with the same buffer and was pasted to the intestine with a fingertip. The whole assembly was put in the dissolution vessel paddle type before adding 250 ml of phosphate buffer (pH 6.6) at a temperature of 37 ± 0.5°C. The rate of stirring was 50 rpm, which was believed to mimic the buccal cavity environment. The time spent for the film to completely erode or detach from mucosa was recorded as the *in vitro* mucoadhesion time (Hassan et al., 2011).

#### Fourier Transform Infrared Spectroscopy

The Fourier transform infrared (FTIR) spectra of the plan film (F1), the selected containing urea and that containing both urea and miconazole (medicated film) recorded by FTIR spectrophotometer (FTIR- Spectrometer, Tensor 27, Bruker, United States). The disk technique was used. The samples were mixed with spectroscopic grade of potassium bromide and compressed in the form of disks using hydraulic press then subjected to scanning from 4000 to 600 cm^-1^.

#### *In Vitro* Release Studies

The release studies were carried out using USP rotating paddle dissolution test apparatus at 37°C and stirring rate of 100 rpm. The medium of dissolution consisted of 200 ml PB (pH 6.8). The later was added to ensure sink conditions. Buccal film (4 cm^2^) which theoretically contains one or more MIC of miconazole base was added to the dissolution media. About 5 ml of the sample was withdrawn at predetermined time intervals (5, 10, 20, 30, 40, 50, 60, 80, 100, and 120 min) and replaced with equivalent amount of fresh medium. Additionally, the samples were filtered if necessary and analyzed using UV spectrophotometer at 272 nm. At each time intervals, the amount of drug released was determined. The drug release profile was constructed by calculating the cumulative amount of drug released vs. time (Hiremath et al., 2009).

#### Microbiological Evaluation of the Drug-Containing Films Against *C. albicans*

The antifungal activity of drug-containing films was evaluated using Kirby Bauer disk diffusion method with some modifications (Bauer et al., 1966). Briefly, circular films (1 cm) containing either of miconazole MIC, 10% urea, or a combination of both together (0.0625 mg/L miconazole and 10% urea) in the same film had laid over the surface of Sabouraud agar seeded with 1 ml of 0.5 McFarland *C. albicans* in normal saline. Furthermore, twice, thrice, 4, or 5 times the MIC of miconazole (0.125, 0.25, 0.5, or 1 mg/L, respectively) was tested either alone or in combination with 20, 30, 40, or 50% of urea, respectively. All plates had incubated at 35 ± 2°C for 24 h. Following the incubation time, plates had investigated for the presence of inhibition zones, which measured and expressed in (mm). All experiments were performed in triplicates and the means had calculated as well as the standard deviation.

### Statistics

Reproducibility was achieved through independent repeating of experiments (minimally three times). Expression of data was in the form of mean and standard deviation. One-way ANOVA, was used to detect the significant differences between the groups considering *p* < 0.05. The data were statistically analyzed using IBM SPSS (17.0, IBM, United States).

## Results

The MIC of miconazole against *C. albicans* was determined using microdilution assay. It was found to be 32 ± 0.09 mg/L, which was a high concentration above the break point (≥8 mg/L) and hence considered resistant organism. A dramatic reduction was recorded in the MIC of miconazole from 32 ± 0.09 mg/L when tested alone, to 0.0625 ± 0.04 mg/L when combined with 10% urea against *C. albicans* resulting in FIC of 0.0019. It was also found that urea had no effect on the test yeast when used alone. Moreover, the minimum fungicidal concentration (MFC) of miconazole alone against *C. albicans* was 64 ± 0.11 mg/L. Interestingly, the MFC equaled the MIC when the drug was combined with 10% urea. These results indicated the presence of synergism between urea and miconazole against *C. albicans* as the FIC value was below 0.5.

**FIGURE 1:**
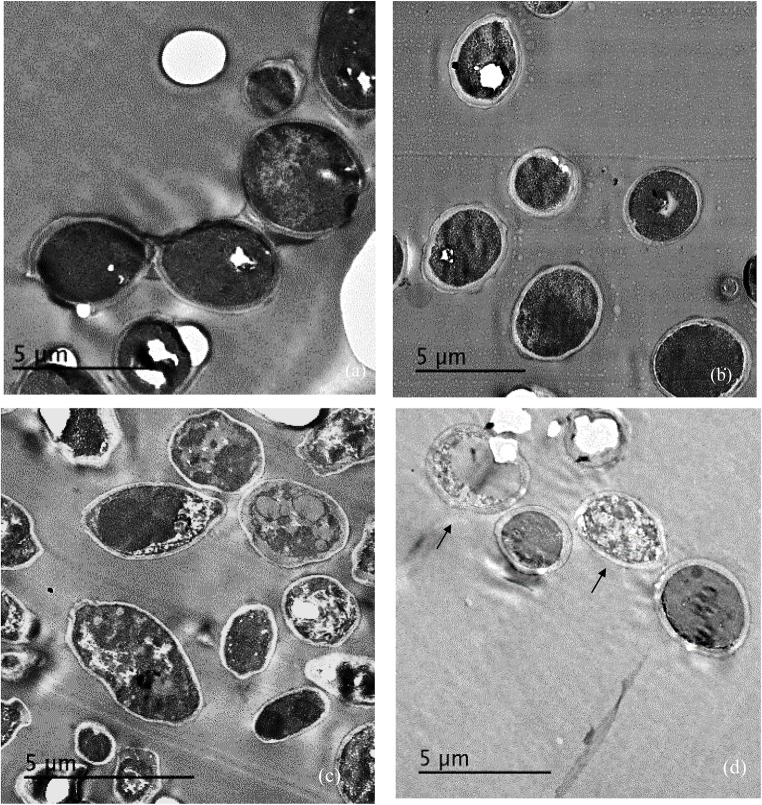
Transmission electron microscope (TEM) images of treated and untreated *Candida albicans* displaying **(a)** a normal morphology with intact cell walls and dense cytoplasm, **(b)** urea- treated cells showing some irregularities in the cell wall but the cytoplasm remained dense, **(c)** miconazole-treated cells with less dense cytoplasm, **(d)** cells treated with a combination of miconazole/urea showing disrupted cytoplasm as pointed by arrows (1200×).

Treatment of *C. albicans* by miconazole in the absence or presence of urea resulted in different morphological changes either at the periphery or inside the cell. This was detected when the treated- as well as untreated-test organism were subjected to transmission electron microscopy. It revealed a dense cytoplasm of the control cells as well as urea treated cells (**Figures 1a,b**). However, the cytoplasmic density was decreased in miconazole-treated cells and markedly reduced in urea/miconazole exposed *C. albicans* cells (**Figures 1c,d**).

**FIGURE 2:**
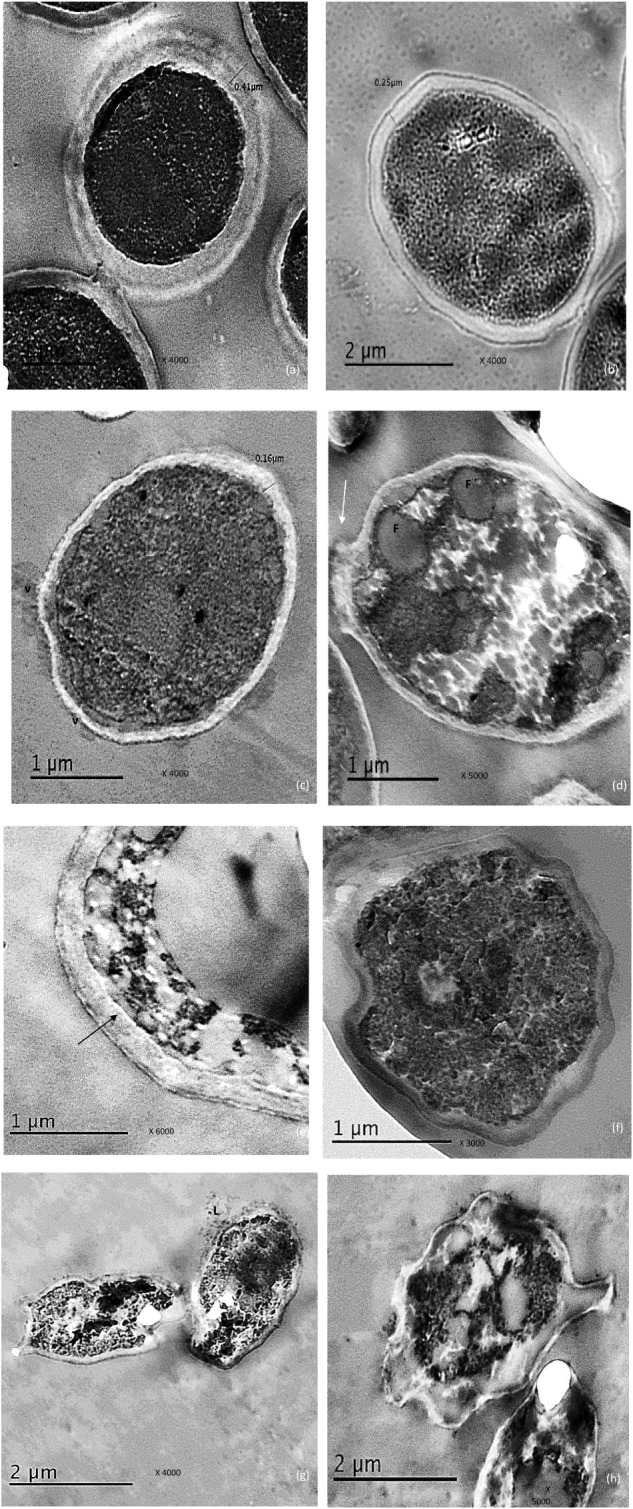
Electro micrographs of *C. albicans* cells examined by TEM showing, **(a)** the control cells with thick, intact cell and dense cytoplasm, **(b)** reduced cell wall thickness in urea –treated cells, **(c)** Thin cell wall as well membrane–limited vesicles (V) representing remnants of destroyed cells when exposed to miconazole, **(d)** some fat deposits (F), damage in the cell wall (arrow) and disrupted cytoplasm were observed in miconazole treated cells, **(e)** disruption in the contact between the cell wall and the inner membrane in miconazole treated cells (arrow), **(f)** early apoptotic cell with condensed chromatin for urea/miconazole treated cells, **(g)** leakage (L) was clearly detected in combination exposed cells (arrow), **(h)** necrotic cells with damaged cytoplasm in combination treated cells. The magnification power was written on each photo.

**Figure 2a** showed a normal morphology of the untreated control cells of *C. albicans* was displayed showing thick cell wall measuring 0.41 μm, intact inner plasma membrane and dense cytoplasm but organelles cannot be distinguished.

Concerning urea treated cells; slightly irregular cell wall and inner plasma membrane were noticed. Moreover, the thickness of the cell wall was reduced to 0.25 μm (**Figure 2b**).

For miconazole treated cells, very thin cell wall measuring 0.16 μm was recorded. There were some membrane-limited vesicles which mostly cytoplasmic remnant of the damaged *C. albicans* cells. These vesicles were found sticking to or in the vicinity of intact cell walls (**Figure 2c**). Some fat globules were detected in the cytoplasm (**Figure 2d**). In addition, some cells showed degradation of the cell wall as shown in **Figure 2d**. Moreover, the contact between the inner plasma membrane and the cell wall was disrupted (**Figure 2e**).

Regarding the subjection of test cells to a combination of miconazole and urea, the shape of the cells had drastically changed showing more irregularities in the cell wall. At low fungicidal dose (0.016 mg/L of miconazole and 10% urea), early enlarged apoptotic cells with condensed chromatin at the periphery of the nucleus was noticed in addition to small blebs that appeared in the form of protruded parts of the cell membrane (**Figure 2f**). At high fungicidal dose (0.031 mg/L of miconazole and 10% urea), necrosis was common showing less dense cytoplasm mostly granular in form with less or no evidence of structured internal organelles. Leakage was clearly observed in addition to more damage and rupture of some cells mostly necrotic cells were also noticed in **Figures 2g,h**.

**Table 1** represented the composition of different formulae of miconazole buccal film after solvent casting. From the table, it could be noticed that F1 is the basic film forming components. F2 to F6 are containing urea with a regular increasing order. F7 to F11 are medicated buccal films containing the antifungal drug and the penetration enhancer also in one MIC increasing order. All films prepared were transparent, uniform, smooth and flexible.

**Table 1:** Composition of different formulae of miconazole buccal films.

Formulae	Miconazol	Urea	CMC	TW	PG
	mg/cm^2^	mg/cm^2^	mg/cm^2^	mg/cm^2^	mg/cm^2^
F1	0.00	0.00	7.5	13	20.2
F2	0.00	0.2	7.5	13	20.2
F3	0.00	0.4	7.5	13	20.2
F4	0.00	0.6	7.5	13	20.2
F5	0.00	0.8	7.5	13	20.2
F6	0.00	1.0	7.5	13	20.2
F7	0.19	0.2	7.5	13	20.2
F8	0.38	0.4	7.5	13	20.2
F9	0.57	0.6	7.5	13	20.2
F10	0.76	0.8	7.5	13	20.2
F11	0.95	1.0	7.5	13	20.2

*F1–F11 representing different formulae composition, CMC is carboxymethyl cellulose, TW is tween 80 and PG is propylene glycol.*

The physical properties of the plan film (F1) was compared to those containing regular increasing amounts of urea (F2–F6) and the others containing increasing amounts of urea/miconazole combination (F7–F11) as presented in **Table 2**. It was noticed that the plan film weight recorded lower values than the theoretical one. Additionally, there was an association between the decrease in the films weight and the marked reduction in the actual drug content. The film thickness increased by increasing the concentration of urea and decreased by increasing miconazole concentrations. The recorded values of different parameters including weight and thickness were comparable to what reported on miconazole films (Rasool and Khan, 2010). The values of the SD for all variables were low indicating the reproducibility of the preparation method. Furthermore, the folding and endurance showed the flexibility of the films which was observed from the capability of the films to tolerate folding several times with no cracking. The palatability of the films could be concluded since the pH of microenvironment of different batches ranged from 6.59 to 6.87. The bio-adhesion strength and time for all medicated films were nearly constants as recorded in (**Table 3**). It also noted that there was no association between bioadhesion time and the bioadhesion strength (**Table 3**). This is due to their dependence on different factors including; the dissolution rate of the film and electrostatic interaction, respectively (Habib et al., 2010).

**Table 2:** Physicochemical properties of the prepared buccal films.

Formulae	Weight (mg/cm^2^)	Folding endurance	Thickness (mm)	pH	Drug content (mg/4cm^2^)	Swelling index
F1	132.86 ± 1.41	>200	0.240	6.59	0.000	2.824 ± 0.064
F2	135.59 ± 0.71	>200	0.275	6.69	0.000	2.798 ± 0.160
F3	137.88 ± 0.58	>200	0.280	6.74	0.000	2.857 ± 0.133
F4	138.68 ± 1.24	>200	0.270	6.78	0.000	2.851 ± 0.125
F5	138.69 ± 1.66	>200	0.280	6.84	0.000	2.836 ± 0.110
F6	136.47 ± 1.46	>200	0.265	6.86	0.000	2.819 ± 0.042
F7	137.00 ± 0.98	>200	0.210	6.74	0.645 ± 0.008	2.832 ± 0.023
F8	138.67 ± 0.59	>200	0.210	6.78	0.774 ± 0.02	2.853 ± 0.051
F9	138.50 ± 1.29	>200	0.209	6.81	0.929 ± 0.04	2.848 ± 0.033
F10	139.33 ± 1.68	>200	0.204	6.85	1.300 ± 0.12	2.778 ± 0.098
F11	138.99 ± 2.41	>200	0.207	6.87	1.560 ± 0.23	2.834 ± 0.088

**Table 3:** Bio-adhesion parameters of the prepared medicated buccal films.

Formulae	Bio-adhesion strength (g)	Adhesion force (*N*)	Bond strength (Nm^-2^)	Bio-adhesion time (min)
F7	2.47 ± 1.99	0.025	5.33	54.2 ± 10.01
F8	2.31 ± 2.10	0.029	5.18	57.6 ± 8.22
F9	2.42 ± 1.87	0.026	5.02	53.7 ± 12.6
F10	2.39 ± 1.92	0.028	5.21	58.3 ± 9.71
F11	2.40 ± 1.73	0.026	5.28	55.4 ± 8.58

**FIGURE 3:**
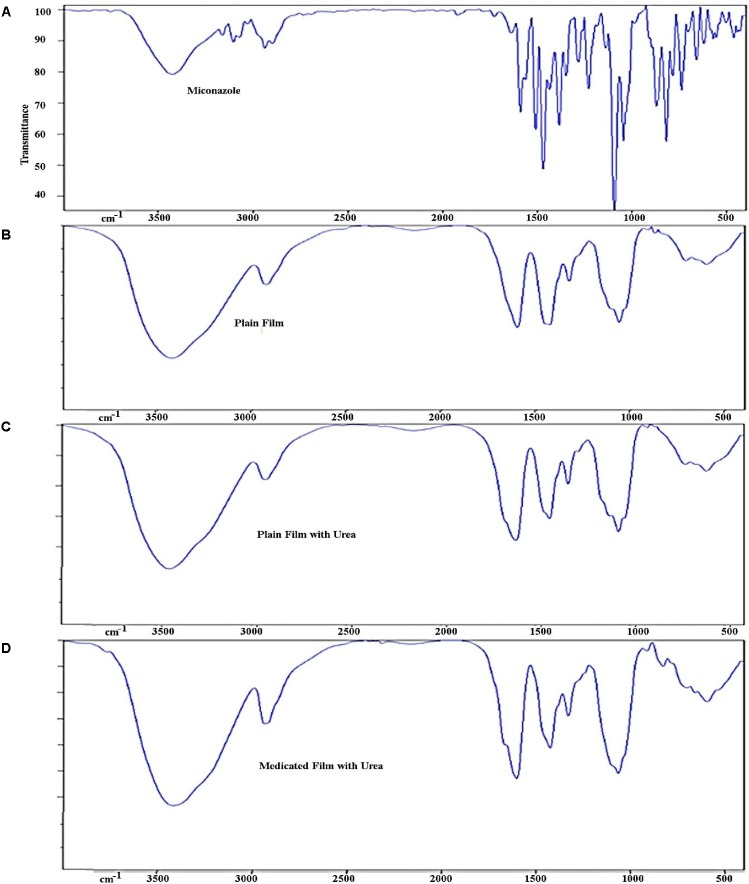
Infra-red spectrum of different prepared films showing **(A)** peaks of miconazole base, **(B)** peaks of plan film components without miconazole, **(C)** plan film containing urea, and **(D)** medicated film containing miconazole. Peaks of miconazole became less intense due to overlapping caused by other ingredients ilm.

Infra scan of the drug showed all of the characteristic peaks of the compound (**Figure 3**). Comparing IR scan of different films, it could be concluded that the peaks of the plan film containing urea led to disappearance of all characteristic peaks of the drug. Subsequently, the entrapment of the drug in the medicated film led to change in the total symmetry of the drug molecules in the polymer molecules.

**FIGURE 4:**
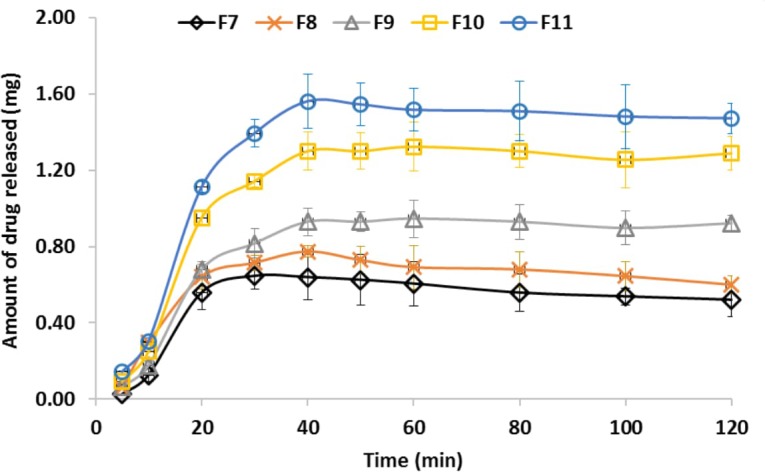
Miconazole release profile from different medicated buccal bioadhesive films. It could be arranged as follows; F11 > F10 > F9 > F8 and F8 = F7. The bars represent the standard deviation.

*In vitro* drug release profile from different prepared medicated films was carried out at conditions simulating the buccal cavity (**Figure 4**). It was found that drug release rate was dependent on the actual amount of drug in the medicated films which could be arranged as follows; F11 > F10 > F9 > F8 and F8 = F7. On applying student *t*-test, there was no significant difference between F7 and F8 (*p* > 0.05) while F9, F10, F11 showed significant differences compared to F7 (*p* < 0.05). The maximum amounts of the drug release in all cases were after 40 min which also equal to the actual amount of drug entrapped in the prepared buccal film. It was found that addition of propylene glycol to the prepared films resulted in increasing the release rates of miconazole. This could be explained with respect to the humectant nature of the propylene glycol which, led to hydration of the films. The rate of drug release was proportional to the amount of water absorbed into a film (Rasool and Khan, 2010).

The kinetics of drug released from different medicated films (F7–F11, their compositions were described in **Table 1**) were summarized in **Table 4**. Linear regression was performed and the correlation coefficient (*R*^2^) values were recorded and used for determination of the best fit to zero-order, first-order or Higuchi diffusion model. The release pattern followed a first- order kinetics revealed a dependence on drug concentration. Higuchi diffusion system could be achieved as in CMC/PVP ibuprofen film (Perioli et al., 2004).

**Table 4:** Kinetics of miconazol release from different formulae according to different kinetic models, and the release efficiency.

		Medicated film^∗^				
		F7	F8	F9	F10	F11
Zero order	*R*^2^	0.830	0.859	0.919	0.919	0.927
	*K*_0_	0.019	0.020	0.026	0.036	0.043
First order	*R*^2^	0.952	0.995	0.975	0.975	0.976
	*K*_1_	0.075	0.089	0.050	0.050	0.046
Higushi	*R*^2^	0.929	0.966	0.975	0.975	0.973
	*K*_H_	0.131	0.141	0.176	0.246	0.295

*^∗^(F7–F11) are medicated films and their compositions described in **Table 1**.*

**FIGURE 5:**
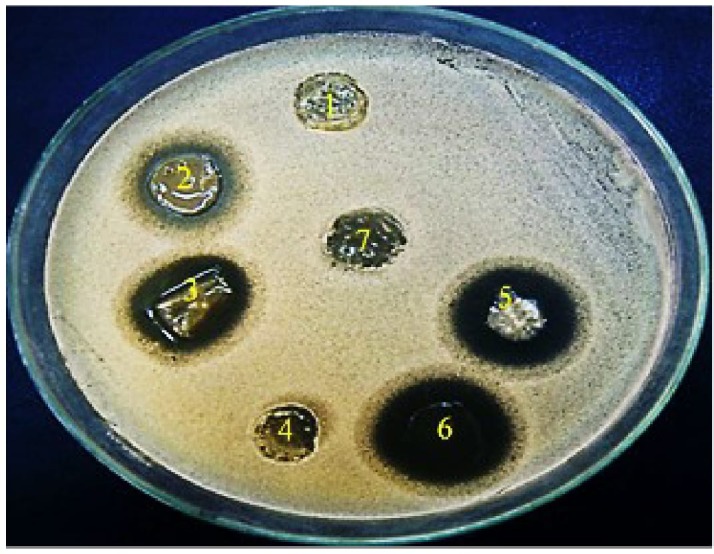
Antifungal activity of miconazole-containing polymer in the absence and presence of urea showing inhibition zones against *C. albicans* around the rounded films. (1) 10% urea, (2) MIC of miconazole (32 mg/L), (3) combination of 10% urea and 0.0625 mg/L of miconazole, (4) plan film, (5) 20% urea and 2MIC of miconazole (0.125 mg/L), (6) combination between 30% urea and 3 MIC of miconazole (0.25 mg/L).

Regarding the evaluation of the antifungal activity of the polymer films, the fungicidal concentration (64 mg/L) of miconazole showed inhibition zone diameters of 18 mm while no zone was formed around film contained miconazole at its newly determined MIC (0.0625 mg/L). Following combination with urea, the increase in the inhibition zone diameters was in a concentration dependent manner. Interestingly, the diameters of the zones were increased significantly (*p* = 0.037) up to 30–40 mm when miconazole/urea combination films were tested (**Figure 5** and **Table 5**). Furthermore, the polymer films were stable and did not melt, by experiment, at the incubatory temperature (37°C) for 24 h as shown in **Figure 5**.

**Table 5:** Evaluation of the growth inhibition of *C. albicans* by drug-containing film using disk diffusion assay.

Test organism	Inhibition zone diameters in (mm)^∗^														
	Group 1			Group 2			Group 3			Group 4			Group 5		
	U 10%	M_1_	U 10% + M_1_	U20%	M_2_	U 20% + M_2_	U30%	M_3_	U 30% + M_3_	U40%	M_4_	U 40% + M_4_	U50%	M_5_	U 50% + M_5_
*C. albicans* (MTCC 227)	0 ± 0	18 ± 0.11	30 ± 0.08	0 ± 0	27 ± 0.22	32 ± 0.08	0 ± 0	30 ± 0.02	35 ± 0.03	0 ± 0	32 ± 0.22	38 ± 0.08	0 ± 0	34 ± 0.08	40 ± 0.05

*^∗^U, urea; M, miconazole; each experiment was carried out in triplicates and the average was calculated ± standard deviation (SD). M1; MIC, M2; twice MIC, M3; 3 times MIC, M4; 4 times MIC, M5; 5 times MIC of miconazole.*

## Discussion

In pharmaceutical technology, urea is one of the well-known penetration enhancers. It could promote transdermal permeation by enhancing hydration of the stratum corneum through inducing the formation of hydrophilic diffusion gates within the barrier (Singh and Choudhury, 2007). In addition, urea is a perfect keratolytic substance that is commonly used with the antifungal agents for treatment of mycoses to enhance the penetration of these drugs in the lesion sites (Martin et al., 2003). Therefore, it was interesting to study the role of urea on the penetration of miconazole through the *C. albicans* cell wall and, consequently the MIC and MFC. Our work showed that *C. albicans* was resistant to miconazole base and the MIC was 32 mg/L. This means high concentration of miconazole will be used to treat the yeast infections and hence will be associated with many side effects. Combining 10% urea with miconazole showed marked decrease in the MIC (0.0625 mg/L) which means 512-fold reduction. Moreover, reduction of MIC to 0.001 mg/L when 20% urea was used indicated highly effective combination (data not shown). Paniagua et al. (2002) reported higher MIC value (50 mg/L) of miconazole against *C. albicans* isolated from the throat of non-AIDS patients. However, Rai et al. (2017) reported that the MIC of miconazole against *C. albicans* isolated from patients suffering from vulvovaginitis, was 18 mg/L. This variation in the MIC value might be due to the different chemical nature of miconazole studied beside the test strains and the protocols used. Santos et al. (2017) mentioned that miconazole nitrate combined with a natural product (polygodial) recorded an MIC value of 0.65 mg/L, which was higher than that reported in the presence of urea in our study. Martins et al. (2006) studied the inhibitory effect of 7.5 to 40% urea on dermatophytes *in vitro*. It was found that 87% of the dermatophytes were susceptible to urea at 12.5%, and that only two isolates of *Trichophyton* spp. needed higher concentration of urea to be inhibited. In addition, the study of Tanuma et al. (2001) reported that using urea along with butenafine or lanoconazole could cause earlier improvement of the dermatological symptoms of *Tinea pedis* with hyperkeratotic properties.

To explain the effectivity of urea/miconazole combination against *C. albicans* in our study, transmission electron microscopy was used. Among the several drastic morphological changes observed, the hallmark features of apoptosis and necrosis that were accounted for miconazole/urea combination-induced cell death leading to potent fungicidal action. Previous studies had reported the morphological changes resulted from treatment of *C. albicans* with different antifungal agents using TEM and SEM. Phillips et al. (2003) mentioned that apoptosis was detected in *C. albicans* at low fungicidal doses of amphotericin B while necrosis was common at high fungicidal doses. Some antifungal agents such as caspofungin exerted a severe harmful effect on the cell wall without affecting the cytoplasm by inhibiting (1,3)-β-D-glucan synthesis (Hao et al., 2013). Additionally, De Nollin and Borgers (1977) reported that treatment using miconazole resulted in an accumulation of membranous components in the cell wall, particularly at the site of bud-formation. Moreover, necrosis of *C. albicans* cells had reported by De Nollin and Borgers (1977) when fungicidal doses of miconazole had used. Furthermore, Miconazole could inhibit 14α-demethylase enzyme which is essential for biosynthesis of ergosterol in the cell membranes of the yeast (Piérard et al., 2012). In addition, Cope (1980) reported that fungistatic and fungicidal doses of miconazole interacted directly with the cellular components of *C. albicans* and resulted in an increase in the intracellular K^+^. Furthermore, farnesol modulated the morphogenesis of *C. albicans* through a downregulation of Saps 2, 4, 5, and 6 expressions (Décanis et al., 2011). However, Hultenby et al. (2014) reported that treatment of *C. albicans* with a mixture of propylene glycol, lactic acid and urea resulted in certain ultrastructural changes but without a specific mode of action.

In the current investigation, the medicated buccal adhesion films were prepared containing gradual increasing concentration of one determined MIC of miconazole and urea. For comparison only, plain films containing gradual increasing amount of urea were prepared. The transparency of the films was attributed to the presence of tween 80 as a basic component in all formulae. In addition, the prepared films were plasti films which might be due to adding propylene glycol as a basic component. At the same time, propylene glycol improved the folding, endurance and flexibility of the prepared films. Excess of propylene glycol would remain in the Petri dish allowing the easily remove of the prepared films. However, propylene glycol was found to have a negative effect on the weight of the medicated films. This could be explained by the study of Al-Badr (1972), who reported that miconazole was soluble in propylene glycol and hence this led to a reduction in the actual drug concentration in the medicated films.

The important factor affecting the adhesion of a polymer was the degree of swelling. Normally, the adhesion occurs shortly after the beginning of the swelling of bioadhesive polymers. At this stage, the bonds formed were not strong enough (Yehia et al., 2009). Relaxation of the originally stretched or entangled polymer chains would be occurred as a result of water uptake and consequently, the bio-adhesive sites within the polymer would be exposed. Accordingly, faster swelling of the polymer led to rapid initiation of diffusion in addition to the formation of adhesive bonds. In our work, the swelling index was constant but lower than that reported by El Maghraby and Abdelzaher (2015). These findings indicated that the water absorption power decreased not only as a result of using propylene glycol as a plasticizer (Basu et al., 2010; Anjankumar, 2011) but also due to the presence of polyvinyl alcohol. The later was known to be crystalline in nature which with low hydration and subsequently low swelling rate (Patel et al., 2015). The disappearance of the drug characteristic stretching and bending peaks may be due the multi components of the prepared plan film including; carboxy methylcellulose, polyvinyl alcohol, and propylene glycol. These components were characterized by strong absorption peaks of their stretching and bending functional groups leading to the disappearance of the entrapped miconazole peaks.

The solubility of carboxy methylcellulose and polyvinyl alcohol in the dissolution media with pH 6.8., in addition to the use of propylene glycol as a plasticizer could explain the complete drug released from the medicated films. The initial as well as the rate of drug release were dependent on the concentration of drug and urea used. This was based on the fact that, increasing the drug content lead to increasing the release rate. Additionally, the role urea as a water soluble substance and hydration enhancer should also be considered. The release pattern followed a first- order kinetics revealed a dependence on drug concentration. Depending on the well-known drug release mechanism and the role of each plan film component, we could consider the following: the solubility of the medicated film components led to the release of the drug as the components dissolved in the dissolution media. The presence of propylene glycol which plasticize the polymer chain led to opening and relaxation of the polymer chains and then the drug release (erosion mechanism). The solubility of urea and propylene glycol might lead to drug diffusion to the dissolution medium. In addition, the role of tween 80, which normally used in pharmaceutical formulation as a release enhancer, should also be considered (Basu et al., 2010; Anjankumar, 2011; El Maghraby and Abdelzaher, 2015).

Concerning the evaluation of the antifungal activity of the polymer films, the diameters of the resulted inhibition zones were increased up to 40 mm for films containing miconazole/urea compared to those containing miconazole alone. Santos et al. (2017) reported that miconazole nitrate combined with polygodial showed inhibition zone diameter of 14 mm against *C. albicans*. Quan et al. (2006) reported similar findings.

## Conclusion

Our study documented the effectivity of miconazole/urea combination against *C. albicans, in vitro*, with extremely reduced MIC value. It worth mention that the present study is the first report on the combined inhibitory action between miconazole and urea on *C. albicans* suggesting that it could be used as an adjuvant in the treatment of yeast infections with less side effects. Additionally, this combination was also formulated in a stable bio-adhesive film and hence it is of potential application in the pharmaceutical industry. Further *in vivo* studies are required to prove for the efficacy and safety of the combination based on animal models and clinical trials.

## Ethics Statement

Collection of animal intestines was dependent on the protocol approved by Research Ethics Committee at the Faculty of Pharmacy, Tanta University, Egypt.

## Author Contributions

LA-M and OM conceived the experiments. LA-M, AD, and OM conducted the experiments. OM analyzed the results. All authors wrote and reviewed the manuscript.

## Conflict of Interest Statement

The authors declare that the research was conducted in the absence of any commercial or financial relationships that could be construed as a potential conflict of interest.
